# Development and Characterization of Chitosan/Porcine Small Intestinal Mucosal Protein Composite Film Incorporated with Curcumin to Extend the Shelf-Life of Chicken Breast

**DOI:** 10.3390/foods15173035

**Published:** 2026-08-27

**Authors:** Jinghan Jiao, Penghui Bi, Jinhao Zou, Jingrong Cheng, Jun Cai, Xuping Wang

**Affiliations:** 1 Key Laboratory of Fermentation Engineering (Ministry of Education), Hubei Key Laboratory of Industrial Microbiology, Cooperative Innovation Center of Industrial Fermentation (Ministry of Education & Hubei Province), Hubei University of Technology, Wuhan 430068, China; 2 Key Laboratory of Functional Foods, Guangdong Key Laboratory of Agricultural Product Processing, Sericultural & Agri-Food Research Institute, Guangdong Academy of Agricultural Sciences, Ministry of Agriculture and Rural Affairs, Guangzhou 510610, China

**Keywords:** porcine small intestinal mucosal protein, chitosan, curcumin, active films, meat preservation

## Abstract

The polysaccharide/protein/polyphenol-based composite films were successfully developed by blending chitosan (CS), porcine small intestinal mucosal protein (PSIMP) and curcumin (CUR) for the short-term refrigerated preservation of chicken breasts. The results showed that the addition of 2% CUR increased the elongation at break by 18.4%, and 3% CUR significantly raised the water contact angle from 53.4° to 72.5°. The water vapor transmission rate and oil absorption rate of CS/PSIMP/CUR3 films were reduced by 30% and 64%, respectively. Throughout the entire refrigerated storage period of chicken breast, packaging with the CS/PSIMP/CUR3 composite film decreased bacterial proliferation by 34% relative to the control group. After 7 days of storage, chicken breast packaged with CS/PSIMP/CUR3 film exhibited a total viable count of 5.57 log CFU/g and a total volatile basic nitrogen level of 13.36 mg/100 g, both remaining below the spoilage thresholds of 6 log CFU/g and 15 mg/100 g, respectively. Additionally, the centrifugal loss and cooking loss decreased by 19% and 32%, respectively. Furthermore, CS/PSIMP/CUR film packages significantly reduced the degradation of inosine monophosphate of refrigerated chicken breasts during storage. The proposed strategy would provide suggestions for developing the novel packaging with antimicrobial and barrier properties for the preservation of perishable meat.

## 1. Introduction

The growing significance of global environmental concerns and the swift advancement of the food industry have intensified the conflict between the non-biodegradability of conventional petroleum-based plastic packaging and the necessity for sustainable food preservation [1]. The continuous spread of white pollution not only damages the ecological environment but also poses potential threats to human health [2]; meanwhile, the high loss rate caused by food spoilage has become a key bottleneck restricting the sustainable development of the food industry. Motivated by this background, the development of environmentally friendly and high-performance natural degradable packaging materials was spotlighted and urgently demanded in the fields of materials science and food industry [3]. Natural macromolecules including chitosan, cellulose, starch and lignin have drawn extensive attention as promising alternatives to petroleum-based plastics. These biomaterials feature abundant raw resources, non-toxicity, renewability and full biodegradability in natural environments. However, naturally biodegradable materials normally contain abundant hydrophilic groups, which leads to markedly inferior oxygen and water vapor barrier properties compared with conventional petroleum-based plastics [4]. Nevertheless, their overall performance can be effectively improved via rational blending and chemical modification.

Chitosan (CS), a natural cationic polysaccharide, is widely considered an optimal alternative to conventional petroleum-based plastic packaging materials, owing to its excellent biocompatibility, biodegradability, inherent antibacterial qualities, and effective film-forming capabilities [5]. Chitosan-based composite films can effectively inhibit microbial growth and delay food deterioration, showing great application potential in food preservation, biomedical packaging and other fields [6]. Benefiting from the global low-carbon development concept and strict plastic restriction policies, chitosan composite materials possess broad industrialization prospects and market development space. Nevertheless, the large-scale application of chitosan films is still restricted by inherent defects and technical bottlenecks. The strong hydrophilicity of pure chitosan endows the film with poor water resistance and humidity stability [7]. Under high-humidity and cold-chain storage conditions, the film is prone to water absorption, swelling and structural loosening, resulting in significant deterioration of mechanical strength and barrier performance against oxygen and water vapor [8]. At present, the composite modification has become the core technical approach to optimize the properties of chitosan films. By compounding CS with functional components such as polysaccharides, proteins, nanoparticles, and plant extracts, the advantages of each component can be effectively synergized, resulting in significant enhancements in the mechanical strength, water vapor, and oxygen barrier properties of chitosan films, while simultaneously improving their antibacterial and antioxidant capacities [9]. Furthermore, this can effectively extend the shelf life of food and reduce food spoilage losses [10]. However, poor interfacial compatibility between these additives and the chitosan matrix easily causes micropore defects and uneven film structure, which further weakens barrier properties. Excessive functional fillers also inevitably damage the mechanical stability of composite films [11].

Porcine small intestinal mucosal protein (PSIMP), as a new type of animal-derived protein, is derived from porcine small intestine, a renewable natural resource, and has high economic value and potential applications [12,13]. Currently, porcine small intestinal mucosa is used for the extraction of heparin, but a large amount of waste is generated during the heparin extraction process [14]. Such waste is rich in mucin but has not been fully utilized, and most of it is directly discharged or treated as low-value feed, resulting in serious resource waste. PSIMP contains a variety of bioactive peptides, which can regulate the body’s inflammatory response, inhibit the expression of pro-inflammatory factors, exert anti-inflammatory effects by targeting macrophages, and also have good biocompatibility and degradability [15]. Recently, natural proteins and polypeptides have emerged as ideal bio-modifiers for chitosan modification, owing to their renewable origin, non-toxicity, excellent interfacial compatibility, and unique biological activity [16]. Different from conventional synthetic additives, proteins and polypeptides can interact with chitosan chains via hydrogen bonding, electrostatic interaction, and hydrophobic crosslinking, effectively optimizing film microstructure, reducing internal micropore defects, and improving structural uniformity [17]. Moreover, inherent antioxidant and antibacterial properties of protein/polypeptide components can endow composite films with enhanced active packaging performance, which effectively inhibits microbial proliferation and lipid oxidation of perishable foods [18]. Therefore, adding PSIMP as a modified component to chitosan films can not only realize the high-value utilization of waste and reduce resource waste but also further optimize the functional properties of composite films, which is a modification method with both environmental significance and economic benefits. Despite the above advantages, the application of protein- and polypeptide-modified chitosan films still faces prominent challenges. Most natural proteins and polypeptides exhibit high hydrophilicity, which inevitably weakens the water resistance and humidity stability of composite films under high-humidity conditions [19]. Excessive protein incorporation may also induce molecular aggregation and phase separation, resulting in deteriorated mechanical strength and barrier performance.

Plant polyphenols can form stable hydrogen bonds and electrostatic cross-linking with chitosan molecular chains, effectively optimizing the internal microstructure of films, reducing micropore defects, and improving structural compactness and mechanical stability [20]. Benefiting from abundant phenolic hydroxyl groups, plant polyphenols possess outstanding antioxidant, antibacterial and free radical-scavenging capacities, which can significantly enhance the active preservation performance of CS composite films. Curcumin (CUR) contains an α,β-unsaturated β-diketone structure belonging to low-molecular-weight phenolic compounds [21,22]. Recently, the multifunctional biological activities of CUR, such as antioxidant, anti-cancer, and antibacterial activities, have been studied extensively and in depth [23]. Moreover, due to its natural safety, it is widely used in the field of active food packaging as a natural antibacterial and antioxidant additive. Active food packaging can achieve film performance enhancement or function expansion by adding bioactive substances, such as endowing films with antioxidant, antibacterial, and pH-indicating functions [24]. CUR has become an ideal choice for developing packaging materials with both active and intelligent properties due to its multiple functionalities [25]. Herein, it is hypothesized that PSIMP and CUR can improve the physicochemical properties of CS films via physical or chemical interactions, enhance their mechanical and barrier performances, and effectively prolong food shelf life by exerting the antioxidant and antibacterial activities of CUR.

Therefore, the present study developed a functional polysaccharide/protein/polyphenol-based composite film, using CS as the substrate, PSIMP as the modified component, and CUR as the functional active component. Moreover, PSIMP was used as a modifier for CS films, which not only provides a utilization direction for porcine small intestinal processing waste but also offers a novel approach for the modification of CS films. Additionally, the coordinated modification of PSIMP and CUR was adopted in this study to achieve the synchronous improvement of the physicochemical properties and functional activities of CS films. The effects of different CUR concentrations on the microstructure, physicochemical properties, and antioxidant activity of the composite films were systematically determined, and their practical application effects in reducing loss and preserving freshness of chicken breast meat were investigated.

## 2. Materials and Methods

### 2.1. Materials and Chemicals

Curcumin (CUR, purity ≥ 98%), inosine monophosphate (IMP), and hypoxanthine (Hx) were obtained from Shanghai Yuanye Biotechnology Co., Ltd. (Shanghai, China). Chitosan (CS, CAS: 9012-76-4, deacetylation degree ≥ 95%, viscosity: 100–300 mPa·s, molecular weight: ~150 kDa) was sourced from Shanghai Yien Chemical Technology Co., Ltd. (Shanghai, China). Papain (≥10 units/mg protein) was sourced from Sigma-Aldrich (Shanghai) trading Co., Ltd. (Shanghai, China). Other reagents were purchased from Tianjin Fuchen Chemical Reagent Co., Ltd. (Tianjin, China). Chicken (Jiangcun Yellow Chicken, 75 days) was purchased from Guangzhou Jiangfeng Industry Co., Ltd. (Guangzhou, China).

### 2.2. Preparation of PSIMP

The PSIMP was prepared by enzymatic hydrolysis. Briefly, a certain amount of intestinal mucosal tissue was dissolved in phosphate-buffered solution (pH = 6), followed by high-speed homogenization at 10,000 r/min for 1 min. Papain was then introduced to start enzymatic hydrolysis, which was performed under optimized conditions of a hydrolysis duration of 6.5 h, an enzyme dosage of 5400 U/g, and a solid–liquid ratio of 4 mL/g. After enzymatic hydrolysis, the enzyme was deactivated by heating in a boiling water bath for 5 min to stop the reaction. The hydrolysate was centrifuged at 4000 r/min for 15 min, after which the supernatant was collected and freeze-dried. Finally, the prepared intestinal mucosal protein powder was stored in a desiccator for subsequent experiments. All hydrolysis parameters were kept constant during PSIMP preparation in this study.

### 2.3. Preparation of CS/PSIMP Films

The preparation of CS/PSIMP films was performed according to a modified method reported by Wen et al. [26]. CS was dissolved in a 1% acetic acid aqueous solution at a 2% concentration, then magnetically stirred at 60 °C for 20 min until a uniform liquid formed. PSIMP was dissolved in water to achieve a 5% (*w*/*w*) concentration and stirred magnetically at 60 °C for 20 min to ensure uniformity. Various CUR solutions were formulated by dissolving CUR at mass fractions of 0, 1, 2, and 3% (*w*/*w*, relative to the combined dry weight of CS and PSIMP) in 1 mL of acetic acid to achieve optimal dispersion.

**Figure 1:**
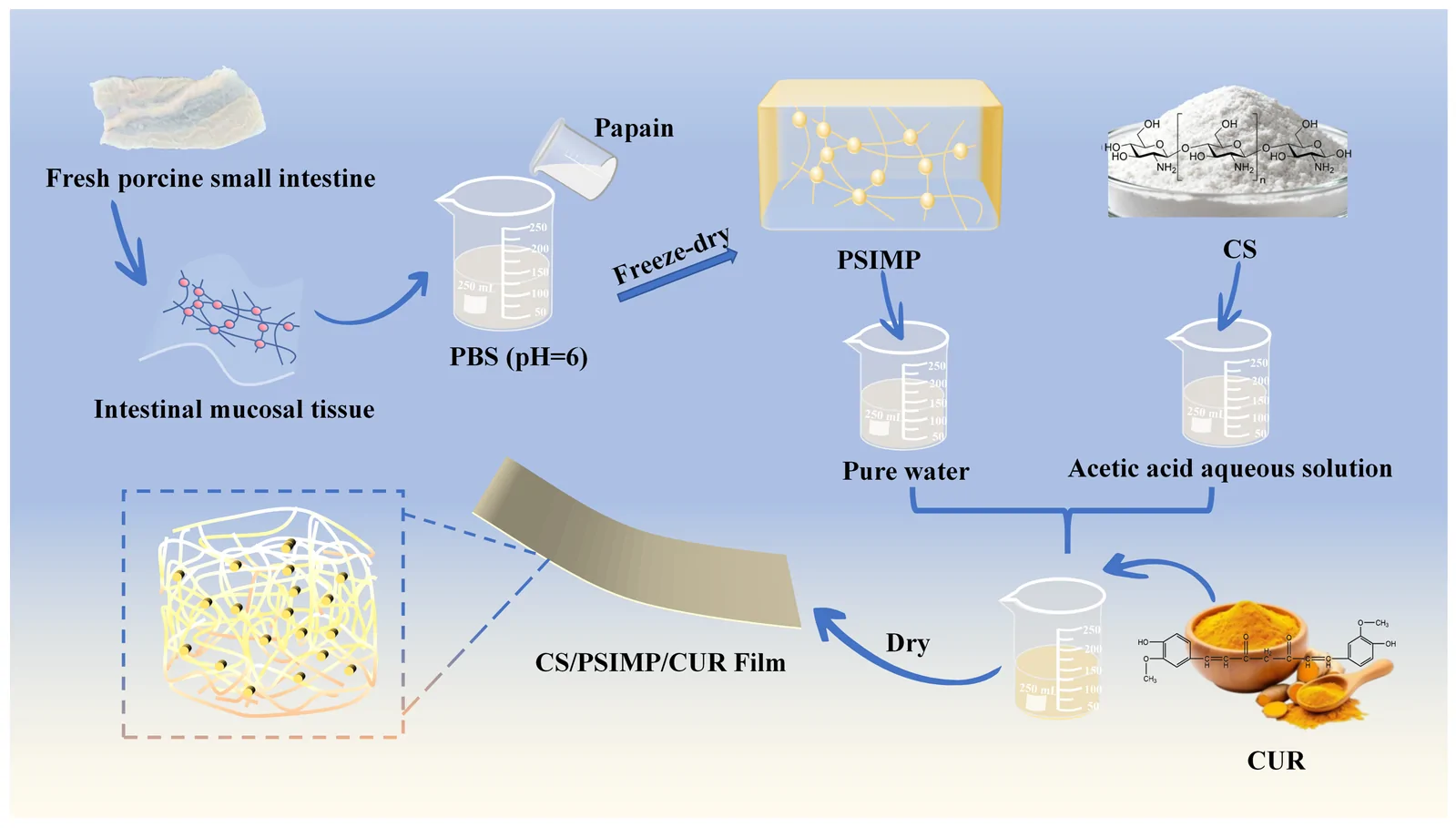
The preparation steps of CS/PSIMP/CUR films.

The prepared CS and PSIMP solutions were combined in a 4:1 mass ratio, and CUR solutions at varying concentrations (0, 1, 2, and 3% *w*/*w*, relative to the total dry weight of CS and PSIMP) were subsequently added. The mixtures were labeled into distinct groups, namely CS/PSIMP, CS/PSIMP/CUR1, CS/PSIMP/CUR2, and CS/PSIMP/CUR3. Subsequently, the mixtures were homogenized at 10,000 r/min for 40 s using a homogenizer and then degassed at 40 °C for 30 min to remove air bubbles. Afterwards, 50 g of the homogeneous CS/PSIMP/CUR film-forming solution was cast into disk containers and dried in an oven at 42 °C for 48 h. Subsequently, the films were kept at a relative humidity of 53% and equilibrated at 25 °C for 2 days (Figure 1).

### 2.4. The Physical Properties of CS/PSIMP/CUR Films

#### 2.4.1. Thickness and Mechanical Properties

The thickness of the films was measured at random positions using a digital micrometer. The tensile strength (TS) and elongation at break (EB) of the films were evaluated by an intelligent electronic tensile testing machine. The films were cut into strips with a size of 60 × 20 mm^2^ and fixed on the grips of the tensile testing machine. The films underwent mechanical fracturing at a strain rate of 1 cm/min with a gauge length of 25 mm, during which force (N) and strain were measured. Each sample underwent quintuple testing, with results represented by the mean value.

#### 2.4.2. Water Contact Angle (WCA)

The hydrophobicity of the films was assessed at room temperature using an OCA40 tester to measure the WCA [27]. Film samples (20 × 80 mm) were attached to a movable sample stage and leveled horizontally. The WCA was measured by placing a 2 μL water droplet on the surface with a precision syringe, and the process was documented using a camera. The WCA was determined by measuring both sides of the droplet and calculating the average. Measurements were conducted at 23 °C with a relative humidity of 40 ± 5%.

#### 2.4.3. Color

The color changes in the films were captured using a colorimeter to obtain the color parameters L* (lightness), a* (red/green), and b* (yellow/blue). The formulas for calculating the whiteness index (*W*_1_) and yellowness index (*Y*_1_) are as follows:

(1) $$Y_{1}=\frac{142.68\times b^{*}}{L^{*}}$$

(2) $$W_{1}=100-\sqrt{{\left(100-L^{*}\right)}^{2}+{a^{*}}^{2}+{b^{*}}^{2}}$$

#### 2.4.4. Water Vapor Transmission Rate (WVTR)

A 10 cm diameter film was prepared, and its WVTR value was measured using a W413 infrared WVTR analyzer (GBPI, Guangzhou Biaoji Packaging Equipment Co., Ltd., Guangzhou, China). Before testing, films were pre-conditioned for 48 h at 25 °C and 53% RH. The effective test area of the instrument test cell was 50.24 cm^2^ and the test temperature was set to 38 °C.

#### 2.4.5. Moisture Content (MC), Swelling Degree (SD), and Water Solubility(WS)

Square film specimens (20 × 20 mm) were weighed to record their wet mass (*M*_1_), followed by oven-drying at 105 °C to constant weight for the measurement of dry mass (*M*_2_). The dried films were placed in a 100 mL beaker with 50 mL of dH_2_O, sealed, and kept at 25 °C for 24 h. Afterward, the films were removed and their surfaces were dried with filter paper and then weighed to determine *M*_3_. The film samples were re-dried at 105 °C until constant weight to acquire the final mass (*M*_4_). Moisture content, swelling degree and water solubility were determined using the formulas listed as follows:

(3) $$MC\left(\%\right)=\frac{M_{1}-M_{2}}{M_{1}}\times 100$$

(4) $$SD\left(\%\right)=\frac{M_{3}-M_{2}}{M_{2}}\times 100$$

(5) $$WS\left(\%\right)=\frac{M_{2}-M_{4}}{M_{2}}\times 100$$

*M*_1_ denotes the film’s initial weight, *M*_2_ its initial dry weight, *M*_3_ the weight after 24 h of water immersion, and *M*_4_ the dry weight post-immersion.

#### 2.4.6. Oil Absorption Rate (OAR)

The filter paper was consistently dried at 50 °C until it reached a stable weight. The filter paper and composite film (4 × 4 cm) were sequentially fixed on a glass test tube containing 5 mL of soybean oil, and the glass test tube was inverted for 48 h [28]. In this setup, soybean oil penetrates the filter paper under gravity and is then absorbed by the composite film; the filter paper serves to homogenize oil distribution rather than collecting penetrated oil. The OAR was calculated by the next formula:

(6) $$OAR\left(\%\right)=\left(\frac{M_{6}}{M_{5}}-1\right)\times 100$$

*M*_5_ represents the initial weight of the dried filter paper, while *M*_6_ denotes its weight after 48 h.

### 2.5. Characterization of CS/PSIMP/CUR Films

#### 2.5.1. Microstructure Observation

Composite film specimens were freeze-dried, mounted onto a black sample stub, and coated with a thin gold film via sputtering. The surface and cross-sectional morphologies of the samples were observed using a Hitachi SN-3400 scanning electron microscope (SEM) (Hitachi Ltd., Tokyo, Japan).

#### 2.5.2. Fourier-Transform Infrared Spectroscopy (FTIR) Analysis

FTIR analysis was employed to record vibration spectra of freeze-dried films samples [29]. The measurement parameters were configured as a scanning scope of 4000 to 500 cm^−1^, 16 scanning times, and a resolution of 4 cm^−1^.

#### 2.5.3. X-Ray Diffraction (XRD) Analysis

The experiment followed the methodology outlined by Ibeogu et al. [28] with slight modifications. An X-ray diffractometer operated at 30 kV and 10 mA was utilized. The diffraction angle was configured to 2θ, covering a scanning range of 5 to 80° at a speed of 15.6°/min.

#### 2.5.4. Differential Scanning Calorimetry (DSC) Analysis

The films’ melting temperature (*T*_m_) was assessed by differential scanning calorimetry (DSC) using a DSC 214 Polymer calorimeter (NETZSCH Scientific Instruments Trading (Shanghai) Co., Ltd., Shanghai, China). The sample, weighing around 3 mg, was analyzed under a nitrogen atmosphere by heating from 0 to 150 °C at a rate of 10 K·min^−1^.

### 2.6. Chicken Breast Preservation Trials

#### 2.6.1. Preparation of Chicken Breast Samples

After slaughter, the chicken breast meat was rapidly transported to the laboratory under cold chain. The fat and connective tissues in the meat were removed, and the chicken meat was randomly cut into slices with a thickness of 2 cm. The sliced chicken breast samples were randomly assigned to four groups, each packaged with film types of CS/PSIMP, CS/PSIMP/CUR1, CS/PSIMP/CUR2, and CS/PSIMP/CUR3. The chicken breast samples were stored at 4 °C under refrigeration. Samples were collected on days 0, 1, 3, 5, and 7 for determinations of TVC, cooking loss, centrifugal loss, IMP and Hx. For TVB-N measurement, extra sampling was conducted on day 9 to monitor the late-stage spoilage evolution of chicken breast.

#### 2.6.2. TVB-N

The TVB-N content was determined with reference to the micro-diffusion method described by Park et al. [30]. A 5 g sample was mixed with 20 mL of distilled water, homogenized, soaked for 30 min, and filtered. Then, 1 mL of the filtrate was introduced into the outer chamber of the diffusion dish, with dH_2_O used for the blank group. A total of 1 mL 20 g/L boric acid and one drop of ethanol-based indicator (containing 1 g/L methyl red and 5 g/L bromocresol green) were added to the inner chamber of the diffusion dish. The lid was partially covered to retain a gap for subsequent addition of 1 mL saturated potassium carbonate solution. After adding the solution, the ground glass lid was pressed flat to seal the diffusion dish. The diffusion dish was incubated at 37 °C for 2 h. After cooling, the inner chamber was titrated with 0.01 M HCl until a blue-purple color appeared. TVB-N values were determined according to the formula below:

(7) $$TVB-N\left(\frac{\mathrm{mg}}{100\;g}\right)=\frac{\left(V-V_{0}\right)\times 14\times c\times 100\times a}{m}$$

where *V* represents the volume of HCl used by the sample (mL), *V*_0_ denotes the volume of HCl used by the blank group (mL), *c* is the concentration of HCl (M), *a* is the dilution factor, and *m* is the sample weight (g).

#### 2.6.3. Determination of TVC

In a sterile setting, 5 g of chicken breast meat was wrapped in film, placed into a sealed food-grade plastic bag to prevent cross-contamination and external interference, and then refrigerated at 4 °C for 7 days. Samples were collected on alternate days for testing. TVC was determined according to China’s National Food Safety Standard (GB 4789.2-2016 [31]) for Food Microbiological Examination-Determination of Total Viable Count. The total bacterial count of the samples was determined in accordance with ISO 4833-1:2013 [32].

#### 2.6.4. Centrifugal Loss

The centrifugal loss was evaluated utilizing the methodology described by Rinwi et al. [33] with minor adjustments. Initially, filter paper was employed to exclude moisture from the surface of chicken breast meat. The central meat pieces (5 ± 0.5 g) were cut, weighed (*M*_7_), wrapped in absorbent paper, and placed in a 50 mL centrifuge tube with absorbent cotton. Following centrifugation at 4000× *g* for 10 min at 4 °C, the meat sample’s surface moisture was removed using filter paper before weighing (*M*_8_). The centrifugal loss of chicken breast meat was calculated by the following equation:

(8) $$Centrifugal\;loss\left(\%\right)=\frac{M_{7}-M_{8}}{M_{7}}\times 100$$

#### 2.6.5. Cooking Loss

The cooking loss was assessed following the procedure outlined by Xu et al. [34], with slight modifications. First, filter paper was used to remove moisture from the surface of the chicken breast meat. Then, the meat was cut into cubes of 4.0 × 3.0 × 3.0 cm^3^ and weighed (*M*_9_), followed by being placed into a vacuum cooking bag. The chicken breast samples were sous-vided cooked at 71 °C for 35 min and subsequently cooled in tap water for 30 min. After blotting the surface moisture with filter paper, they were reweighed (*M*_10_). Each group consisted of three chicken breast cubes, from which three subsamples were extracted for the experiment. The cooking loss of chicken breast meat was calculated by the following equation:

(9) $$Cooking\;loss\left(\%\right)=\frac{M_{9}-M_{10}}{M_{9}}\times 100$$

#### 2.6.6. IMP and Hx

Sample preparation followed the method outlined by Zou et al. [35]. A total of 3 g of sample was combined with 15 mL of 10% cold perchloric acid, homogenized at 10,000× *g* for 2 min, and then centrifuged at 10,000× *g* for 15 min at 4 °C. The supernatant was collected, and the precipitate was re-extracted with another 15 mL of 10% cold perchloric acid. The supernatants were merged, and the pH was adjusted to 6.5–6.8 using sodium hydroxide solutions of 0.5 M and 0.05 M concentrations. Samples were incubated at 4 °C for 30 min to precipitate perchloric acid, followed by centrifugation at 10,000× *g* for 15 min at the same temperature. A 1 mL aliquot of the supernatant was filtered using a 0.22 μm membrane filter. According to the method reported by Liu et al. [36], high-performance liquid chromatography (HPLC) was employed with a ZORBAX SB-C18 column (250 mm × 4.6 mm, 5 μm) and an ultraviolet detector set at 254 nm for analysis. Chromatographic conditions included a column temperature of 30 °C, an injection volume of 20 μL, and a flow rate of 0.8 mL/min. The elution program lasted 25 min, utilizing 98% 0.05 mol/L potassium dihydrogen phosphate buffer (mobile phase A) and 2% methanol (mobile phase B). The mobile phases were prepared by filtering through a 0.45 μm membrane and sonication before use. The contents of IMP and Hx were qualitatively and quantitatively determined based on the retention times and standard curves of the corresponding reference standards.

### 2.7. Statistical Analysis

All tests were conducted with parallel replicates: five replicates for mechanical properties (thickness, TS, EB), and three replicates for other film characterization and meat freshness indicators. All results were presented as mean ± standard deviation. One-way analysis of variance (ANOVA) followed by Duncan’s multiple-range test was adopted to detect significant differences, and statistical analyses were carried out using IBM SPSS Statistics 26 with the significance level set at *p* < 0.05. Lowercase letters in film-related figures and uppercase letters in meat storage figures represent significant differences between different formulations and storage days, respectively. All figures were plotted with Origin 2021 software.

## 3. Results and Discussion

### 3.1. The Physical Properties of CS/PSIMP/CUR Films

#### 3.1.1. Thickness, Mechanical Properties, and WCA

**Figure 2:**
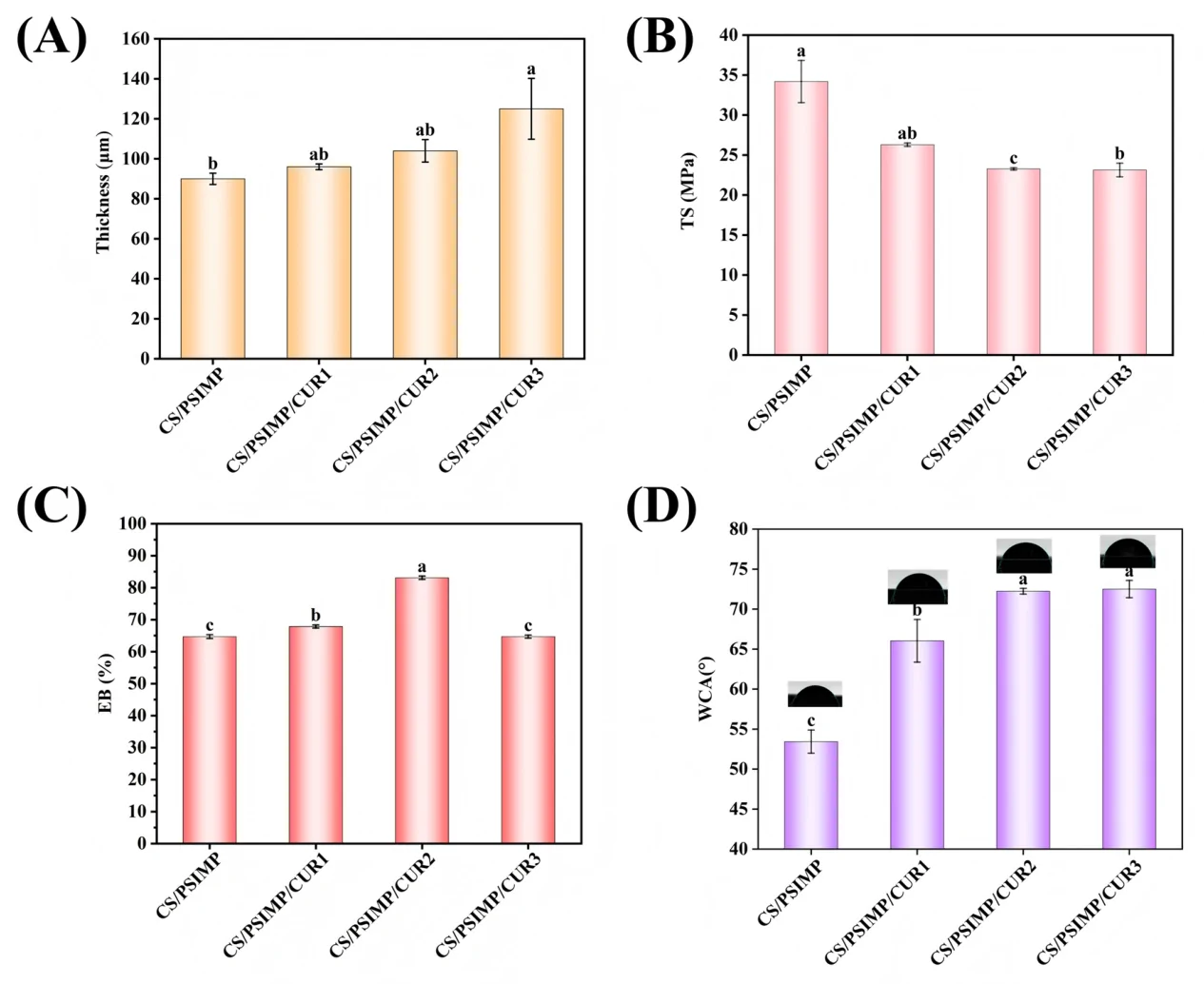
The thickness (**A**), mechanical properties (**B**,**C**), and WCA (**D**) of CS/PSIMP-based films with different CUR contents. The error bars represent the standard deviation between replicates of the same experiment. Different letters show significant differences among the films (*p* < 0.05).

As shown in Figure 2, the film thickness increased from 90 to 120 μm with increasing CUR concentration from 0 to 3%, while significant differences were only detected between the CS/PSIMP and CS/PSIMP/CUR3 films. This could be attributed to the rise in solid content [37]. Similarly, Yu et al. [38] observed that incorporating proanthocyanidin-loaded nanocomplexes significantly enhanced the thickness of films composed of chitosan and chondroitin sulfate.

TS represents the maximum stress that a film can withstand under tensile loading, whereas EB indicates the maximum deformation of the film prior to fracture. The TS and EB values of CS/PSIMP films with different concentrations of CUR were shown in Figure 2B,C. The CUR-free CS/PSIMP film exhibited the highest TS of 34.2 MPa and the lowest EB of 64.7%. Increasing the CUR content to 2% resulted in a significant decrease in TS to 23.3 MPa and an increase in EB to 83.1% (*p* < 0.05). This indicated that CUR acts as a plasticizer, enhancing the film’s mechanical properties by reducing intermolecular binding forces, such as van der Waals forces, and increasing polymer chain flexibility [39]. When the CUR content was further increased to 3%, the TS remained at 23.1 MPa, and the EB decreased to 64.7%, possibly because CUR aggregation intensified mutual friction and ultimately impaired the compactness of the film [40].

In meat processing and preservation, the hydrophobicity of films directly determines their ability to block water, prevent adhesion, and resist condensation, which are key properties for maintaining meat quality and extending shelf life. The water contact angle (WCA) assesses the hydrophobicity of composite films, with values below 65° indicating hydrophilicity and values above 65° indicating hydrophobicity [26,41]. Figure 2D shows the WCA of CS/PSIMP-based films with different CUR concentrations. The CS/PSIMP film lacking CUR exhibited the minimum WCA value of 53.4°. Incorporation of CUR significantly increased the WCA of film. The WCA values of CS/PSIMP/CUR1, CS/PSIMP/CUR2, and CS/PSIMP/CUR3 films were 66.0°, 72.2°, and 72.5°, respectively. These findings demonstrated the hydrophobic nature of the films containing CUR. This may be attributed to the hydrophobic properties of CUR, which reduced the probability of water molecules adhering to the film surface [42]. Another reason could be due to addition of CUR enhancing the interaction between CS and PSIMP within the film matrix, while simultaneously reducing the number of exposed hydrophilic groups, thereby increasing the hydrophobicity of the film surface [43].

#### 3.1.2. Color

**Figure 3:**
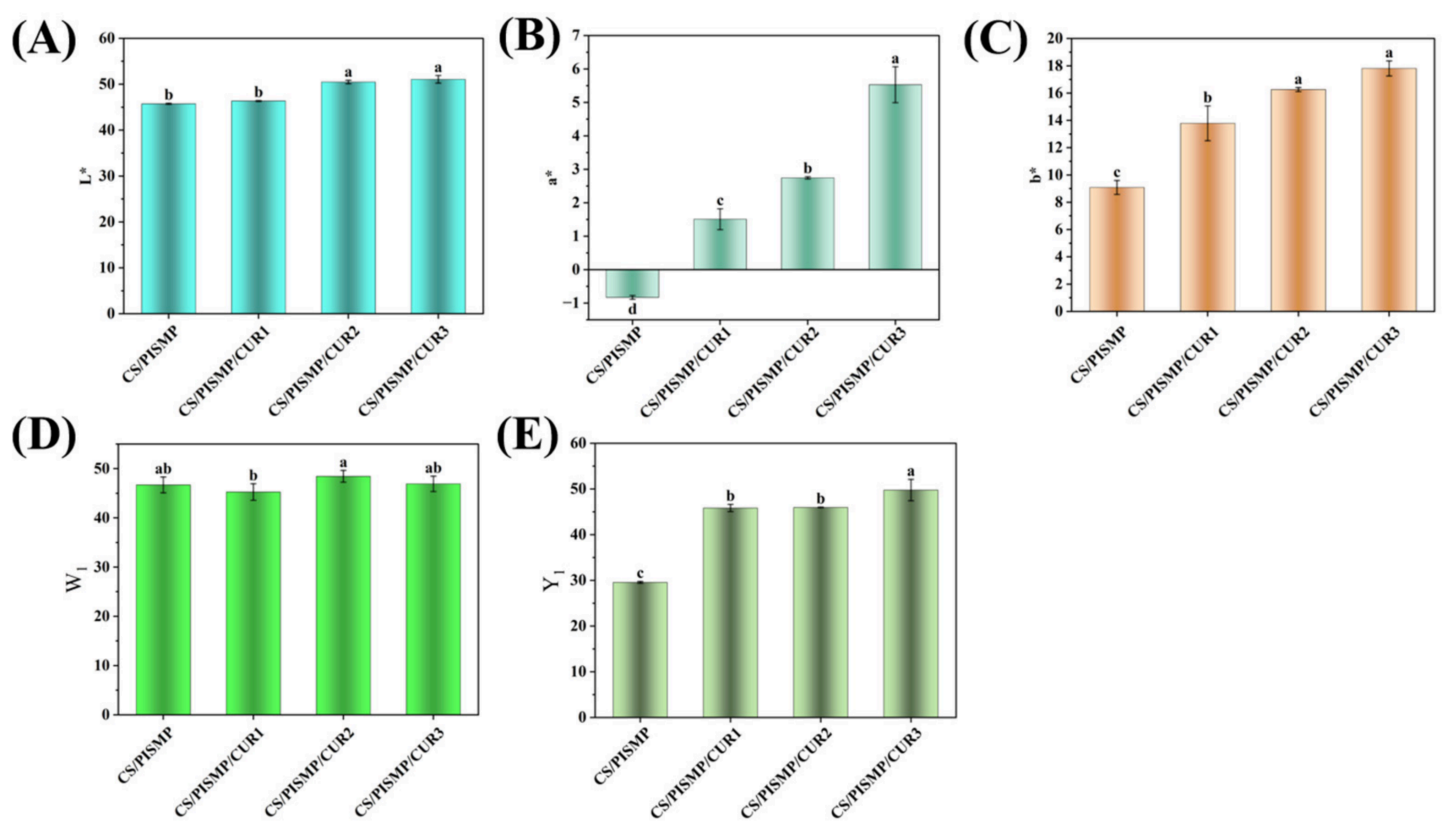
Color of CS/PSIMP-based films with different CUR contents. Lightness (L*) (**A**); Red-green chromaticity (a*) (**B**); Yellow-blue chromaticity (b*) (**C**); Whiteness index (*W*_1_) (**D**); Yellowness index (*Y*_1_) (**E**). The error bars represent the standard deviation between replicates of the same experiment. Different letters show significant differences between the films (*p* < 0.05).

The color parameters (Lightness (L*), Red-green chromaticity (a*), Yellow-blue chromaticity (b*)) of films are shown in Figure 3. With the increase in CUR content, the L* values gradually increased (Figure 3A). Meanwhile, the films containing 2% and 3% CUR exhibited significantly higher (*p* < 0.05) L* values compared with the control and CS/PSIMP/CUR1 films. This increase in lightness was mainly attributed to hydrogen-bonding interactions between CUR and the CS/PSIMP matrix, which optimized the inner microstructure of films and enhanced internal light scattering behavior. For a* values, the control film displayed a negative a* value, while CUR addition converted a* from negative to positive with a continuous increasing trend, and the film containing 3% CUR achieved the maximum a* value (Figure 3B). Similarly, b* rose significantly with elevated CUR dosage, which resulted in an intensified yellow hue (Figure 3C). Such a color shift originated from the intrinsic orange-yellow pigment property of CUR, which was homogeneously dispersed throughout the polymeric network and endowed the resultant films with yellow-orange appearance. With the increase in CUR content, the whiteness index *W*_1_ (Figure 3D) of each experimental group showed no significant difference compared with the control group without CUR. This may be because yellow CUR increases the b* value and tends to reduce the *W*_1_. Meanwhile, CUR alters the internal pores and phase structure of the film to enhance light scattering and raise the L* value, increasing *W*_1_. These two effects counteract each other, so no significant difference in *W*_1_ exists between CUR-added groups and the control group. However, the yellowness index *Y*_1_ (Figure 3E) gradually increased with the increase in CUR loading. With the rise in CUR content, *Y*_1_ increased from 29.57 to 49.77. This phenomenon may be related to the inherent yellow property of CUR.

#### 3.1.3. WVTR and OAR

**Figure 4:**
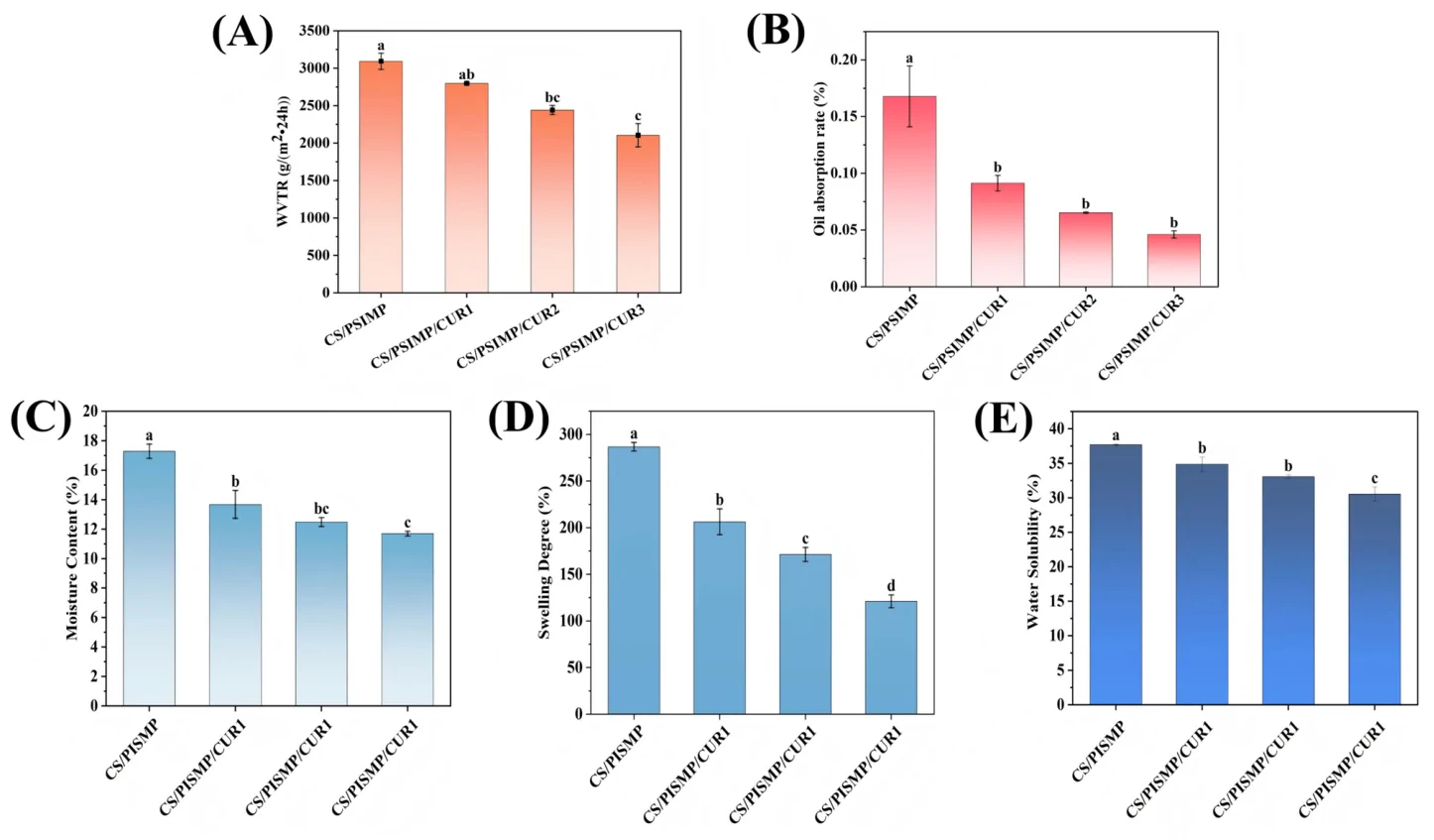
WVTR (**A**), OAR (**B**) and water sensitivity (**C**–**E**) of CS/PSIMP-based films with different CUR contents. The error bars represent the standard deviation between replicates of the same experiment. Different letters show significant differences between the films (*p* < 0.05).

The WVTR and OAR can be used to evaluate the barrier performance of film, which is an important indicator related to the shelf life of packaged foods. The CS/PSIMP film had the highest WVTR value. With increasing CUR content in the films, the WVTR value slightly decreased (Figure 4A). The possible reason was the hydrophobic properties of CUR, which effectively repelled water vapor and thus improved the water barrier performance of composite films [44]. Additionally, the formation of dense H-bonds between CUR and the film matrix might reduce intermolecular chain space and lower WVTR [45].

The OAR indicates the oil resistance capacity of CS/PSIMP films. As displayed in Figure 4B, the CS/PSIMP film showed the highest OAR value. As the CUR content in the films increased, the OAR value decreased from 0.167 to 0.046%. No significant differences were observed when the CUR concentration ranged from 1% to 3% (*p* > 0.05). This phenomenon might be attributed to CUR filling the film pores, altering the interactions between polymer chains, and reducing the porosity and specific surface area of the film [46]. At the same time, the hydrophobicity and crosslinking effect of CUR weakened the adsorption and accommodation capacity of the film toward the oil phase. Therefore, higher CUR loading allowed more pores to be filled, and the oil absorption rate continued to decrease.

#### 3.1.4. Water Sensitivity

The water sensitivity of films is generally characterized by three principal parameters: moisture content, swelling rate, and solubility [47]. This property is of paramount importance for food packaging and preservation applications. Moisture content is a crucial indicator of film performance, since it directly influences the chemical and physical properties of the films and microbial growth within the packaged system. Films with reduced moisture content frequently exhibited enhanced structural integrity and dimensional stability [48]. As presented in Figure 4C, the CS/PSIMP composite film displayed significantly higher moisture content relative to other film formulations (*p* < 0.05). Moreover, a gradual reduction in film moisture content was observed with the incremental incorporation of CUR.

The water resistance of composite films is notably affected by changes in their swelling ratio and solubility [28]. Figure 4D,E illustrated the swelling ratio and solubility characteristics of CS/PSIMP films loaded with various concentrations of CUR. Incorporation of CUR was found to effectively improve the water resistance of CS/PSIMP films, as evidenced by the simultaneous reduction in both thew swelling ratio and solubility. The inherent swelling behavior of CS and PSIMP macromolecules was primarily attributed to the abundant hydrophilic hydroxyl groups. Importantly, the reduction in the solubility of CS/PSIMP/CUR films exhibited a distinct dependency on the CUR loading level. Within the CS/PSIMP/CUR polymer matrix, CUR established strong hydrogen-bonding interactions with the hydroxyl and other hydrophilic functional groups of the CS/PSIMP polymers. The formation of hydrogen bonds limited the mobility of water molecules within the polymer chains, resulting in reduced solubility of the composite films. These findings were consistent with those reported by Ibeogu et al. [28].

### 3.2. Characteristics of Films

#### 3.2.1. Morphological Images

**Figure 5:**
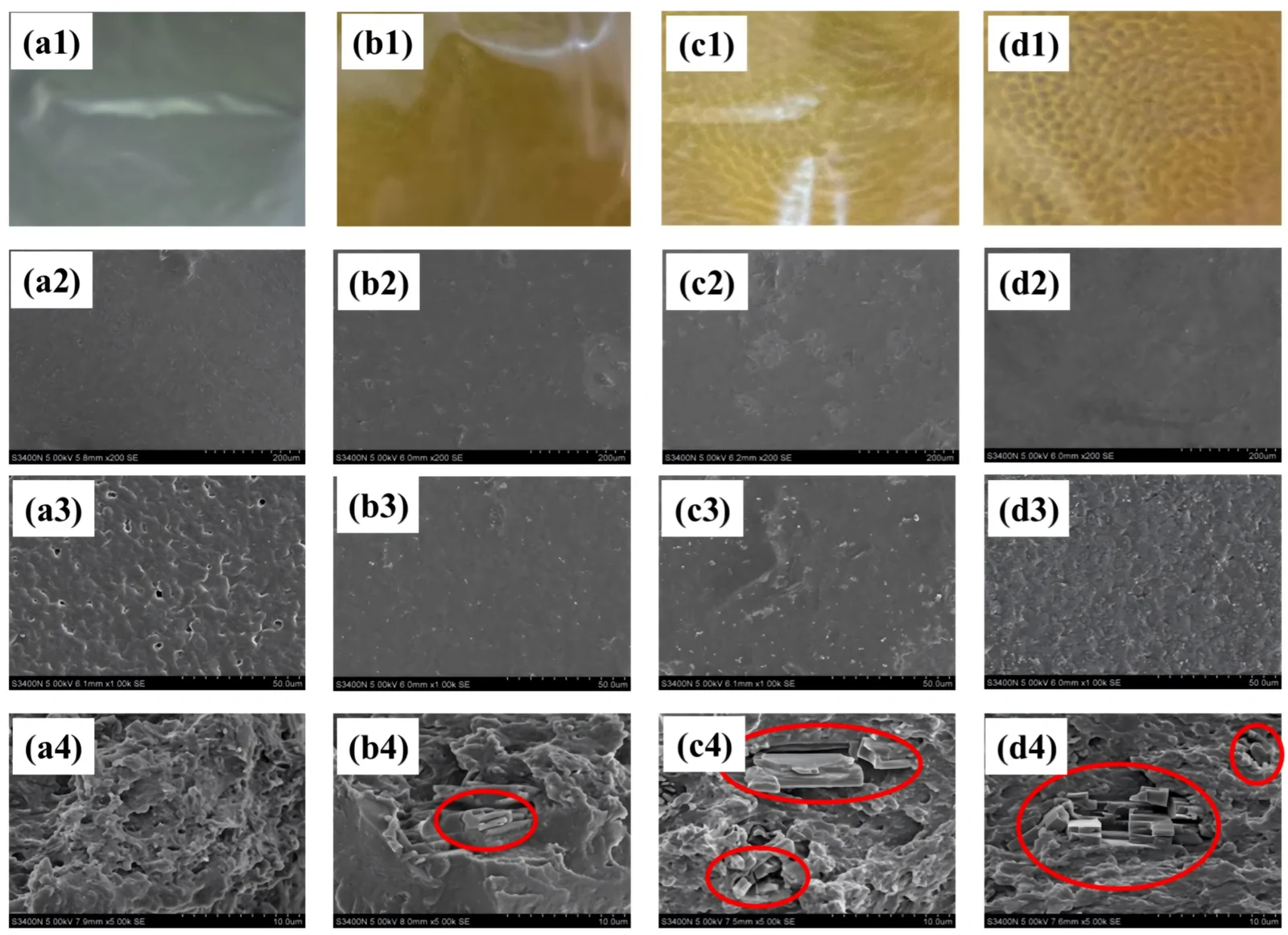
The images of (**a1**–**d1**) were the appearance of CS/PSIMP, CS/PSIMP/CUR1, CS/PSIMP/CUR2, and CS/PSIMP/CUR3 composite films, respectively. The images of (**a2**–**d2**) and (**a3**–**d3**) were the surface morphology of the films at magnifications of 200× and 1000×, respectively. The images of (**a4**–**d4**) were the cross-sectional morphology of the composite film at a magnification of 5000×. Red circles indicate CUR crystal aggregation in cross-section SEM images.

Morphological images of films with different CUR contents observed by digital camera and SEM at magnifications of 200× and 1000× are presented in Figure 5. Visual observation from digital photographs demonstrated that all films remained macroscopically intact without visible large cracks. Nevertheless, SEM cross-section micrographs revealed microscale pores and voids, as well as tiny internal cracks within pure CS/PSIMP at the microscopic level. The CS/PSIMP film without CUR was colorless and transparent. After the addition of 1% CUR, the film gradually turned pale yellow. Furthermore, increasing the CUR concentration to 2% and 3% deepened the film color (Figure 5(a1–d1)). At the same time, obvious aggregations were observed on the surfaces of CS/PSIMP/CUR2 and CS/PSIMP/CUR3 films (Figure 5(a2–d2,a3–d3)). Additional structural analysis was conducted using SEM cross-sectional images at 5000× magnification, as illustrated in Figure 5(a4–d4). The cross-sectional structure of the CS/PSIMP film exhibited an irregular surface with visible cracks and pores. This phenomenon could be ascribed to the macromolecular characteristics of CS and PSIMP. The intermolecular interactions between these two components generated large interstitial voids inside the film matrix. With the incorporation of CUR, the gullies on the cross-section of composite films gradually vanished. Increasing CUR dosage further rendered the cross-sectional microstructure more orderly and compact. Compared with the control, CS/PSIMP/CUR3 exhibited a relatively denser cross-sectional structure with reduced large micropores, though considerable structural heterogeneity and minor local CUR crystal aggregation could still be clearly observed (Figure 5(d4)). Such structural features originated from hydrogen-bonding interactions and the formation of an interpenetrating network between CS and PSIMP. Upon CUR incorporation, these interactions contributed to improved microstructure, yet complete homogeneity of the matrix was not achieved.

#### 3.2.2. FTIR

**Figure 6:**
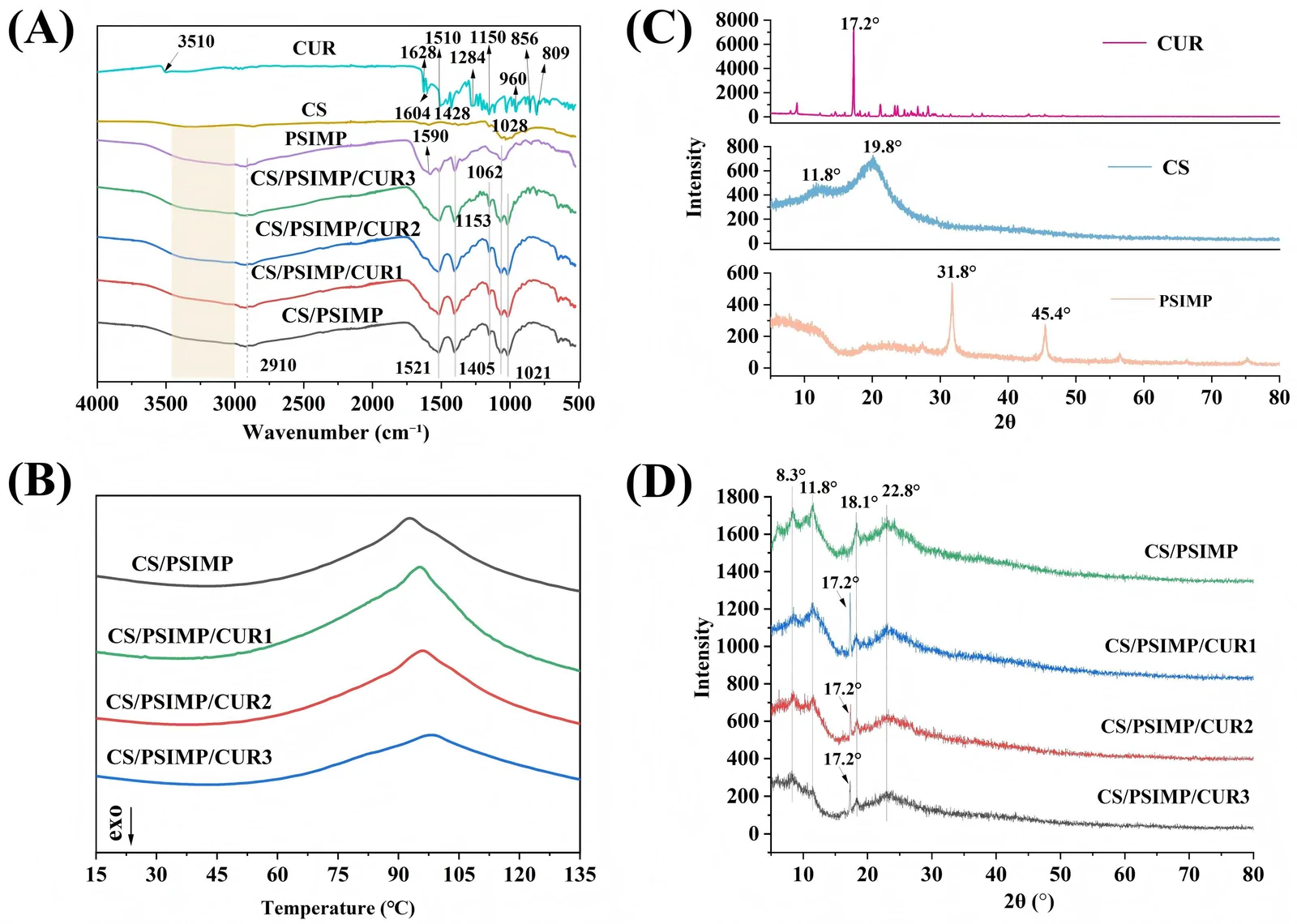
FTIR spectra (**A**), DSC curves (**B**), and the XRD patterns (**D**) of CS/PSIMP-based films with different CUR contents. The XRD patterns (**C**) for CUR, CS and PSIMP.

The molecular interactions within the film matrix were analyzed by FTIR. The spectra of the individual CS, PSIMP and CUR, as well as the CS/PSIMP, CS/PSIMP/CUR1, CS/PSIMP/CUR2, and CS/PSIMP/CUR3, were separately analyzed, as shown in Figure 6A. According to Chen et al. [49], the CUR spectra exhibited a band at 3510 cm^−1^ corresponding to O-H stretching vibration of phenol, and the peaks at 1628, 1604, 1510, 1428, 1284 and 1150 cm^−1^ were attributed to aromatic C=C stretching, benzene ring stretching, C=O and C=C stretching vibrations, olefinic C-H bending vibrations, aromatic C-O stretching, and C-O-C stretching, respectively. According to Liu et al. [12], the PSIMP spectrum exhibited a band at 2922 cm^−1^ corresponding to the C-H stretching vibration of the alkyl group, while peaks at 1579 and 1401 cm^−1^ were attributed to the C-O stretching and N-H bending vibrations of the amide group, respectively. According to Okwuenu et al. [50], the CS spectrum exhibited bands at 1604 and 1428 cm^−1^ corresponding to amide I and carbonyl group vibrations, while the peak at 1028 cm^−1^ was associated with amide II (N-H bending) and primary amine groups. In the spectrum of CS/PSIMP film, a broadened absorption peak at 3000 cm^−1^, corresponding to the stretching vibration of hydroxyl groups. The absorption peaks at 1521 and 1405 cm^−1^ belonged to C=O stretching (amide I) and N-H vibrations in the amide II, and the absorption peaks at 1153 and 1062 cm^−1^ referred to the amide III band [51]. Compared with pure PSIMP powder, obvious variations in band shape and intensity were observed for CS/PSIMP film within the 3000 cm^−1^ region and 1400–1600 cm^−1^ fingerprint region, suggesting the possible establishment of hydrogen-bonding interactions between CS and PSIMP. After CUR was incorporated into the CS/PSIMP matrix, no new distinct characteristic absorption peaks were generated, and most major stretching-vibration bands were largely retained. These observations indicated that physical interactions dominated among CUR, CS, and PSIMP, which were consistent with previous findings [29].

#### 3.2.3. DSC and XRD

The effect of CUR incorporation on the thermal behavior of CS/PSIMP films was analyzed by DSC in Figure 6B. The pure CS/PSIMP film exhibited a broad endothermic peak at 92.8 °C. For hydrophilic polysaccharide–protein composite films, this endothermic event in the range of 90–100 °C is a comprehensive thermal response, which originates from the combined effects of bound water dehydration and macromolecular denaturation, rather than a single structural transformation. After CUR incorporation, the endothermic peak temperatures of CS/PSIMP/CUR1, CS/PSIMP/CUR2, and CS/PSIMP/CUR3 films shifted slightly upward to 95.2 °C, 95.9 °C, and 98.2 °C, respectively. The gradual shift in the thermal transition peak suggests variations in the molecular chain mobility and intermolecular interaction environment within the composite matrix, which may be associated with improved interfacial compatibility after CUR modification [52,53,54].

The crystal structures of pure compounds of PSIMP, CUR, and CS were analyzed by XRD at diffraction angles of 2θ ranging from 5 to 80° (Figure 6C). The characteristic peaks of CUR indicated its highly crystalline structure [55]. The characteristic peak of CS at 11.8° corresponded to a semi-crystalline structure, and a strong diffraction peak near 19.8° belonged to the regular lattice of heterogeneous crystallization [56,57]. PSIMP displayed two sharp characteristic peaks at 31.8° and 45.4°, confirming its crystallinity, which was similar to the results of Tenebrio Molitor protein investigated by Bu et al. [58].

The crystalline properties of the films are presented in Figure 6D. Compared with the XRD spectra of CS and PSIMP, no characteristic peaks appeared at 19.8°, 31.8°, and 45.4°, whereas a new weak diffraction peak at 8.3° was observed in the XRD spectrum of the CS/PSIMP films. These results suggested that PSIMP was compatible with CS, and the blend displayed an amorphous structure [29]. After CUR incorporation, a new diffraction peak at 17.2° was detected in CUR-loaded composite films, and the peak intensity increased gradually with the elevation of CUR dosage, implying the partial existence of crystalline CUR in the film matrix [59]. The incorporation of CUR into the CS/PSIMP film did not markedly alter the XRD pattern, yet reduced the intensities of diffraction peaks at 8.3°, 11.8°, 18.1°, and 22.8°. These subtle variations in peak intensity indirectly indicate the formation of intermolecular hydrogen bonds between CUR and the CS/PSIMP matrix, which slightly disturbs the original molecular arrangement and crystalline order of the CS/PSIMP network [27,28,45]. Consistent with the FTIR results, the XRD data only provides indirect structural evidence for intermolecular interactions, and no definitive molecular structural model is concluded merely based on the spectral and diffraction results.

### 3.3. Application in Real Meat Models

#### 3.3.1. TVB-N

**Figure 7:**
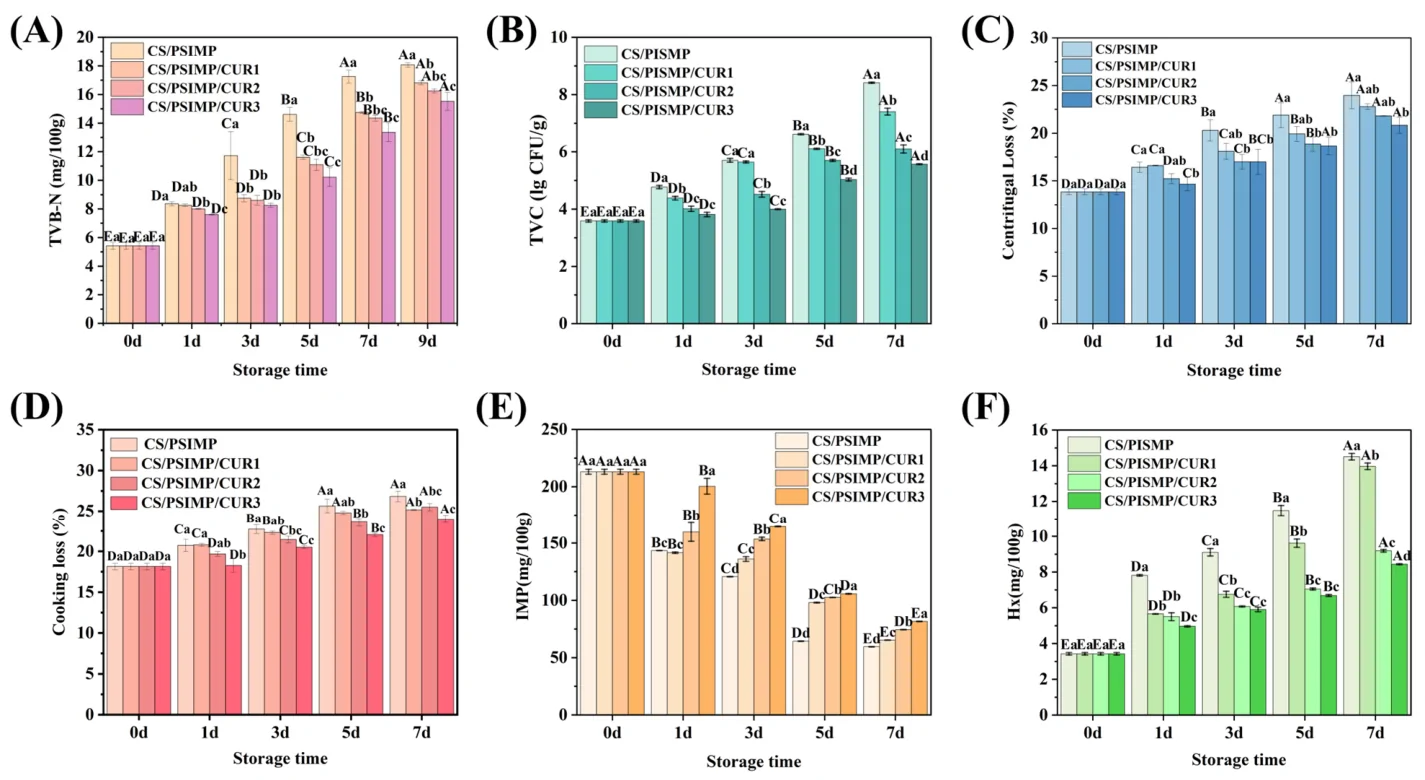
The effect of CS/PSIMP-based films on TVB-N (**A**) and TVC (**B**) of chicken breast. The changes in centrifugal loss (**C**) and cooking loss (**D**) of chicken breast meat during the storage period. The effect of CS/PSIMP-based films on the IMP content (**E**) and Hx content (**F**) in chicken breasts. The error bars represent the standard deviation between replicates of the same experiment. Different uppercase letters indicate significant differences among different film formulations at the same storage time; different lowercase letters indicate significant differences among different storage times for the same film formulation (*p* < 0.05). Note: Day 9 data were only obtained for the TVB-N assay.

The rise in TVB-N content during chilled chicken storage is often linked to bacterial growth and increased endogenous enzyme activity [60]. Consequently, the TVB-N assay serves as a reliable indicator to assess whether chicken breast samples comply with national food safety standards, thereby ensuring the freshness of chilled chicken products. The TVB-N content of chicken breast showed a continuous upward trend as storage time extended. As illustrated in Figure 7A, the initial TVB-N value of chicken breast was approximately 5.43 mg/100 g on day 0. According to the Chinese National Food Safety Standard (Fresh and Frozen Animal Meat Products; GB 2707-2016 [61]), meat is regarded as fresh when its TVB-N content is ≤15 mg/100 g. After 7 days of storage, the TVB-N content in the CS/PSIMP packaged chicken breast rose to 17.26 mg/100 g, surpassing the freshness threshold. The TVB-N levels in chicken breast in the CS/PSIMP/CUR1, CS/PSIMP/CUR2, and CS/PSIMP/CUR3 packaged groups were 14.75, 14.35, and 13.36 mg/100 g, respectively, all staying below the 15 mg/100 g threshold. The CS/PSIMP/CUR3 film-packaged chicken breast exhibited the optimal preservation performance, with the TVB-N content rising to 15.53 mg/100 g after 9 days of storage. In addition, our previous study confirmed that unpackaged chicken breast exhibited a TVB-N value of 18.56 mg/100 g upon 4 days of storage at 4 °C, which exceeded the acceptable limit and was classified as spoiled meat [62]. These findings demonstrated that CS/PSIMP films incorporated with CUR could effectively lower TVB-N accumulation and retard the deterioration of meat products, consistent with previous reports by Mai et al. [23].

#### 3.3.2. TVC

The safety of refrigerated chicken meat is highly correlated with the TVC of the product. As shown in Figure 7B, the initial TVC value of fresh chicken breast samples was determined to be 3.59 log CFU/g. Notably, the CS/PSIMP/CUR1, CS/PSIMP/CUR2, and CS/PSIMP/CUR3 composite packaging effectively inhibited bacterial growth and proliferation in refrigerated chicken breasts throughout storage. After 7 days of refrigerated storage, the TVC value of the group packaged with CS/PSIMP film reached 8.41 log CFU/g. In contrast, the TVC values of chicken breast samples packaged with CS/PSIMP/CUR1, CS/PSIMP/CUR2, and CS/PSIMP/CUR3 films were significantly lower, at 7.41, 6.10, and 5.57 log CFU/g, respectively. These findings demonstrated that the incorporation of CUR could effectively retard the proliferation of spoilage bacteria in chilled chicken breast during storage, thereby prolonging the shelf-life of chilled chicken breast. Previous research has validated the antibacterial efficacy of active films containing CUR, which is consistent with the present results. Khan et al. [63] fabricated active films containing β-cyclodextrin/CUR and observed a remarkable reduction in the TVC of chicken meat, consistent with the antibacterial properties of the CS/PSIMP/CUR composite films identified in this research.

#### 3.3.3. Water Loss

Water loss is a critical indicator for evaluating meat quality, as numerous studies have confirmed a significant negative correlation between water loss and meat quality; lower water loss is associated with better overall meat quality [64]. Accordingly, reducing water loss during meat storage is of great significance, as it can effectively improve the juiciness, tenderness, and overall palatability of meat products and improve their economic value [65]. Centrifugal loss and cooking loss indices are typically employed to evaluate the water-holding capacity of meat [66]. The effects of CS/PSIMP/CUR film packaging on the water loss of chicken breast are presented in Figure 7C,D. Throughout the entire storage period, the chicken breast samples in all four experimental groups showed a gradual increase in water loss and a concomitant decline in water retention capacity.

At the initial storage stage (day 0), the centrifugal loss (Figure 7C) of chicken breast was 13.84%. After 7 days of storage, the centrifugal loss of the CS/PSIMP, CS/PSIMP/CUR1, CS/PSIMP/CUR2, and CS/PSIMP/CUR3 groups increased to 23.97%, 22.79%, 21.82%, and 20.83%, respectively. Statistical analysis revealed that the composite films containing CUR significantly reduced the centrifugal loss values of chicken breast compared with the CUR-free film group (*p* < 0.05). In terms of cooking loss (Figure 7D), relative to fresh chicken breast samples, the cooking loss of the four groups increased by 47.82%, 38.73%, 40.61%, and 32.45% after 7 days of storage, respectively. This trend was consistent with the variation in centrifugal loss over the same storage period. The findings indicated that CUR-loaded composite films effectively retarded muscle protein oxidation in chicken breast during storage, retained immobilized water within muscle tissues and mitigated progressive water loss.

#### 3.3.4. IMP

IMP is universally acknowledged as a pivotal indicator for evaluating the freshness of meat. Nevertheless, IMP is unstable during storage and undergoes degradation into bitter-tasting Hx [67]. Accordingly, the deterioration of meat freshness during storage is intrinsically linked to the hydrolytic degradation of IMP. As depicted in Figure 7E, the IMP content significantly decreased over extended storage periods (*p* < 0.05), indicating a progressive decline in the freshness of chicken breast [68]. After 7 days of storage, the IMP content in the CS/PSIMP group of chicken breasts diminished from 213 to 60 mg/100 g, representing a reduction of 71.8%. In contrast, the CS/PSIMP/CUR1, CS/PSIMP/CUR2, and CS/PSIMP/CUR3 groups showed IMP content decreases of 69.48, 65.26, and 61.66%, respectively.

#### 3.3.5. Hx

Furthermore, over the course of storage, the degradation of IMP leads to the generation of Hx, which consequently impairs the sensory quality of chicken breast meat [69]. With extended storage time, the Hx content in all treatment groups of chicken breast increased significantly (*p* < 0.05), as illustrated in Figure 7F. At the initial storage stage (day 0), the Hx content of fresh chicken breast meat was 3.43 mg/100 g. After 7 days of storage, the Hx content in the CS/PSIMP group rose to 14.51 mg/100 g, whereas the CS/PSIMP/CUR1, CS/PSIMP/CUR2, and CS/PSIMP/CUR3 groups reached 13.97, 9.19, and 8.44 mg/100 g, respectively. These findings demonstrated that packaging chicken breast meat with CUR-containing composite films could significantly reduce the degradation rate of IMP during storage, effectively suppress the accumulation of Hx, and consequently maintain the freshness of chicken breast meat.

## 4. Conclusions

In this study, a multifunctional CS/PSIMP-based film was successfully developed by incorporating CUR. The characterization results suggested that intermolecular hydrogen-bonding interactions occurred between CUR and the CS/PSIMP matrix. Such interfacial interactions slightly disturbed the original crystalline order of CS/PSIMP, leading to changes in the composite network structure. These changes contributed to the improvement in mechanical properties and hydrophobicity of the CS/PSIMP/CUR composite films. The incorporation of 3% CUR significantly modulated WVTR and water sensitivity of the composite film, rendering its barrier properties more suitable for food preservation requirements. Furthermore, the CS/PSIMP/CUR3 composite film could effectively retard the proliferation of spoilage bacteria in chilled chicken breast during storage. During the storage period, this film lowered TVB-N content and moisture loss of chicken breasts, thereby significantly retarding spoilage and improving the freshness and quality stability of the stored chicken. These findings demonstrate that CUR can act as an effective functional modifier for CS/PSIMP active packaging films. Moreover, porcine small-intestinal mucosal protein exhibits promising prospects as a raw material for edible food-packaging films, providing references for the high-value utilization of this by-product and the research-and-development of chitosan-based food-packaging materials.

It should also be noted that CUR was added according to theoretical feeding ratios in this study. The actual retained CUR amount inside composite films was not quantified, and CUR release behavior was not investigated. Therefore, we cannot fully distinguish whether the observed chicken breast preservation effect originates from released CUR, the physical barrier function of the CS/PSIMP matrix, or their combined effects. Further quantification of residual CUR in films and release profile tests are required to clarify the underlying preservation mechanism, which deserves exploration in future research. Furthermore, it also be worth noting that several practical barriers remain before PSIMP-based food films can be implemented in real world food packaging applications. Raw material traceability, the initial microbial load of porcine small intestinal mucosa, residues originating from the extraction process, allergenic potential, and the migration of protein components into food matrices are all urgent issues to be addressed. Regulatory requirements, allergen labeling obligations, religious dietary constraints, consumer acceptance, and industrial scale production feasibility also require comprehensive evaluation.

## Data Availability

The original contributions presented in this study are included in the article. Further inquiries can be directed to the corresponding authors.

## Abbreviations

The following abbreviations are used in this manuscript:

CS	Chitosan
PSIMP	Porcine small intestinal mucosal protein
CUR	Curcumin
IMP	Inosine monophosphate
Hx	Hypoxanthine
TS	Tensile strength
EB	Elongation at break
WCA	Water contact angle
WVTR	Water vapor transmission rate
OAR	Oil absorption rate
TVB-N	Total volatile basic nitrogen
TVC	Total viable count
